# Evaluation of FET PET Radiomics Feature Repeatability in Glioma Patients

**DOI:** 10.3390/cancers13040647

**Published:** 2021-02-05

**Authors:** Robin Gutsche, Jürgen Scheins, Martin Kocher, Khaled Bousabarah, Gereon R. Fink, Nadim J. Shah, Karl-Josef Langen, Norbert Galldiks, Philipp Lohmann

**Affiliations:** 1 Research Center Juelich, Institute of Neuroscience and Medicine (INM-3, -4, -11), 52425 Juelich, Germany; r.gutsche@fz-juelich.de (R.G.); j.scheins@fz-juelich.de (J.S.); m.kocher@fz-juelich.de (M.K.); g.r.fink@fz-juelich.de (G.R.F.); n.j.shah@fz-juelich.de (N.J.S.); k.j.langen@fz-juelich.de (K.-J.L.); n.galldiks@fz-juelich.de (N.G.); 2 RWTH Aachen University, 52062 Aachen, Germany; 3 Department of Stereotaxy and Functional Neurosurgery, Faculty of Medicine and University Hospital Cologne, University of Cologne, 50937 Cologne, Germany; khaled.bousabarah@uk-koeln.de; 4 Center for Integrated Oncology (CIO), Universities Aachen, Bonn, Duesseldorf and Cologne, 50937 Cologne, Germany; 5 Department of Neurology, Faculty of Medicine and University Hospital Cologne, University of Cologne, 50937 Cologne, Germany; 6 Department of Neurology, University Hospital RWTH Aachen, 52074 Aachen, Germany; 7 JARA-BRAIN-Translational Medicine, 52074 Aachen, Germany; 8 Department of Nuclear Medicine, University Hospital RWTH Aachen, 52074 Aachen, Germany; 9 Center for Integrated Oncology (CIO), Universities Aachen, Bonn, Duesseldorf and Cologne, 52074 Aachen, Germany

**Keywords:** machine learning, artificial intelligence, textural features, amino acid PET

## Abstract

**Simple Summary:**

Initial studies suggested the additional diagnostic value of amino acid positron emission tomography (PET) radiomics using the tracer O-(2-[^18^F]fluoroethyl)-L-tyrosine (FET) in brain tumor patient management. However, to ensure the reliable performance of the generated FET PET radiomics models for clinical diagnostics, repeatability of radiomics features is essential. Hence, we assessed the impact of brain tumor volumes and key molecular alterations such as an isocitrate dehydrogenase (IDH) mutation on the repeatability of FET PET radiomics features in 50 newly diagnosed glioma patients. In a test–retest approach based on routinely acquired FET PET scans, we identified 297 repeatable features. The IDH genotype did not affect feature repeatability. Moreover, these robust features were able to differentiate patients with IDH-wildtype glioma from those with an IDH mutation. Our results suggest that robust radiomics features can be obtained from routinely acquired FET PET scans, which are valuable for further standardization of radiomics analyses in neurooncology.

**Abstract:**

Amino acid PET using the tracer O-(2-[^18^F]fluoroethyl)-L-tyrosine (FET) has attracted considerable interest in neurooncology. Furthermore, initial studies suggested the additional diagnostic value of FET PET radiomics in brain tumor patient management. However, the conclusiveness of radiomics models strongly depends on feature generalizability. We here evaluated the repeatability of feature-based FET PET radiomics. A test–retest analysis based on equivalent but statistically independent subsamples of FET PET images was performed in 50 newly diagnosed and histomolecularly characterized glioma patients. A total of 1,302 radiomics features were calculated from semi-automatically segmented tumor volumes-of-interest (VOIs). Furthermore, to investigate the influence of the spatial resolution of PET on repeatability, spherical VOIs of different sizes were positioned in the tumor and healthy brain tissue. Feature repeatability was assessed by calculating the intraclass correlation coefficient (ICC). To further investigate the influence of the isocitrate dehydrogenase (IDH) genotype on feature repeatability, a hierarchical cluster analysis was performed. For tumor VOIs, 73% of first-order features and 71% of features extracted from the gray level co-occurrence matrix showed high repeatability (ICC 95% confidence interval, 0.91–1.00). In the largest spherical tumor VOIs, 67% of features showed high repeatability, significantly decreasing towards smaller VOIs. The IDH genotype did not affect feature repeatability. Based on 297 repeatable features, two clusters were identified separating patients with IDH-wildtype glioma from those with an IDH mutation. Our results suggest that robust features can be obtained from routinely acquired FET PET scans, which are valuable for further standardization of radiomics analyses in neurooncology.

## 1. Introduction

Radiomics, a subdiscipline of artificial intelligence, is based on high-throughput quantitative analysis of routinely acquired imaging data, facilitating the development of mathematical models to support clinical decision-making. Most commonly, image features are usually extracted from predefined volumes-of-interest (VOIs). Importantly, image quality deviations caused by non-standardized acquisition parameters, varying segmentations, or image post-processing steps may considerably affect quantitative radiomics features regarding repeatability and generalizability [1]. Furthermore, feature repeatability may depend on phenotype differences in extracranial tumors [2,3]. Therefore, identifying robust features is essential to ensure the reliable performance of radiomics models for clinical diagnostics [4]. For example, robust image features can be identified using test–retest analyses in phantoms, which are repeatedly examined with the same acquisition protocol. As a result, similar but not identical sets of images generate radiomics features that should yield similar results.

One approach towards reproducible radiomics analyses is the Image Biomarker Standardization Initiative (IBSI) [5]. IBSI provides mathematical definitions for radiomics features that have already been integrated into the most commonly applied radiomics software packages, such as LIFEx [6] or the open-source PyRadiomics package in Python [7]. Adhering to the IBSI recommendations may improve the reproducibility and provide the basis for clinical implementation of the generated models.

Currently, radiomics is attracting increasing attention in medical imaging and also in neurooncology [8]. In particular, the majority of studies demonstrated the potential of radiomics based on magnetic resonance imaging (MRI) for clinical applications in patients with primary and secondary brain tumors, e.g., the prediction of an isocitrate dehydrogenase (IDH) mutation in glioma patients or the differentiation of radiation-induced changes from local tumor recurrence in patients with brain metastases [9,10,11,12,13,14]. Furthermore, efforts in terms of the evaluation of MRI feature robustness and repeatability in brain tumor patients are ongoing [15].

Due to an increasing body of literature, particularly the acceptance and application of amino acid positron emission tomography (PET) in brain tumor patients has steadily increased over the past few years [16,17]. Its value has been demonstrated for various important neurooncological applications [17,18,19,20,21]. Moreover, the Response Assessment in Neuro-Oncology (RANO) Working Group and the European Association of Neuro-Oncology (EANO) recommend amino acid PET as a complementary tool to contrast-enhanced MRI in all disease stages [19,22]. Recently, initial studies have investigated the potential of amino acid PET radiomics using the frequently applied tracer O-(2-[^18^F]fluoroethyl)-L-tyrosine (FET), either alone or in combination with MRI [11,14,23,24]. For radiotherapy planning, FET PET has been confirmed to be valuable for improved target volume definition by delineating the non-enhancing tumor parts, which is of considerable interest especially in glioma patients [25,26,27,28].

Due to the present lack of data, our study’s goal was to evaluate the robustness and repeatability of FET PET radiomics features using a novel dual image reconstruction approach that allows for a test–retest analysis based on clinical FET PET. Furthermore, we evaluated the ability of robust FET PET radiomics features for the differentiation of IDH-wildtype from IDH-mutant gliomas.

## 2. Patients and Methods

### 2.1. Patients

Fifty patients (mean age, 50 ± 15 years; age range, 21–82 years; 17 females, 33 males) with newly diagnosed glioma were retrospectively identified and histomolecularly characterized according to the World Health Organization (WHO) classification of Tumors of the Central Nervous System of 2016 [29]. The glioma diagnoses were distributed as follows: WHO grade IV glioblastoma, IDH-wildtype (*n* = 24); WHO grade IV glioblastoma, IDH-mutant (*n* = 2); WHO grade III anaplastic astrocytoma, IDH-wildtype (*n* = 4); WHO grade III anaplastic astrocytoma, IDH-mutant (*n* = 11); WHO III anaplastic oligodendroglioma, IDH-mutant (*n* = 5); WHO grade II diffuse astrocytoma, IDH-wildtype (*n* = 2); WHO grade II diffuse astrocytoma, IDH-mutant (*n* = 2). In addition to structural MRI, all patients had undergone FET PET. Table 1 presents further details of the patient cohort.

### 2.2. Determination of the IDH Genotype and 1p/19q Co-Deletion

For assessment of the IDH genotype, the IDH1R132H protein expression level was evaluated by immunohistochemistry [30,31]. In the case of negative immunostaining, IDH was directly sequenced. The 1p/19q co-deletion status was analyzed by fluorescence in situ hybridization [32].

### 2.3. FET PET Imaging

The amino acid FET was produced and applied, as described previously [33,34]. All patients underwent a dynamic FET PET scan from 0 to 50 min post-injection of 3 MBq of FET per kg body weight on a high-resolution 3 T hybrid PET/MR scanner (BrainPET, Siemens Medical Systems, Inc., Erlangen, Germany) [35]. Image data were corrected for random and scatter coincidences, as well as dead time and isotope decay, before ordinary Poisson ordered subset expectation maximization (OSEM) reconstruction using software provided by the manufacturer (2 subsets, 32 iterations). The reconstructed dynamic dataset consisted of 16 time frames (5 × 1 min, 5 × 3 min, 6 × 5 min). Since the hybrid PET/MR scanner did not provide a transmission source, attenuation correction was performed by a template-based MRI approach [36].

### 2.4. Dual FET PET Image Reconstruction for Test–Retest Analysis

**Figure 1:**
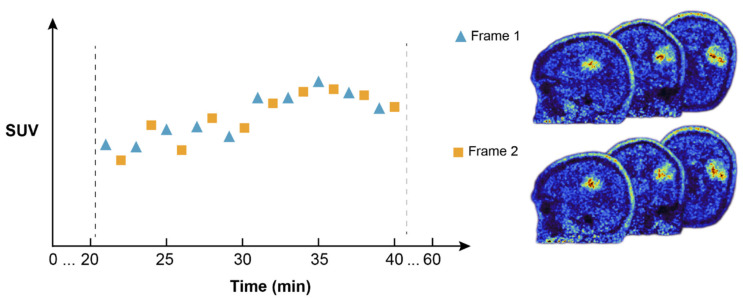
Dual O-(2-[^18^F]fluoroethyl)-L-tyrosine (FET) positron emission tomography (PET) image reconstruction for test–retest analysis. Valid events within 20 to 40 min post-injection of FET were separated in two time windows (frames) and further used for reconstructing the test and retest images. The schematic represents the standardized uptake value (SUV) over time for a single patient. Frame 1 is reconstructed from odd frame events (blue triangles), Frame 2 from even frame events (orange squares), resulting in similar but statistically independent subsamples of PET images (right).

PET data can be stored in list-mode data format containing information about all detected coincidence events in terms of detector numbers in which the photon pair was detected, as well as time stamps. Usually, all coincidence events within a particular time window (frame) are used for image reconstruction. Here, within 20 to 40 min post-injection, only events with odd time stamps were used to reconstruct the test image and only events with even time stamps to reconstruct the retest image. By this dual reconstruction, equivalent but statistically independent subsamples of PET images were created, which are suitable for test–retest analysis (Figure 1).

### 2.5. Image Segmentation for Test–Retest Analysis

The standardized uptake value (SUV) was used to normalize the FET uptake by dividing the radioactivity in the tissue by the radioactivity injected per kilogram of body weight. A spherical VOI of constant size (diameter, 30 mm; volume, 14 mL) was positioned in normal-appearing brain tissue, including gray and white matter, in the contralateral hemisphere. A three-dimensional auto-contouring process using a tumor-to-brain ratio (TBR) of 1.6 or more was used for segmenting the tumor VOI. This threshold is based on a biopsy-controlled study in which this value separated best between vital tumor and healthy brain parenchyma in FET PET [37]. Furthermore, to account for the influence of the limited spatial resolution of PET on the repeatability of radiomics features, spherical VOIs with different sizes (range of volumes, 0.5–33.0 mL; range of diameters, 10–40 mm) were positioned both in the healthy background and centered on the maximum FET uptake in the tumor VOI. All VOIs were defined in the conventionally reconstructed summed PET images from 20–40 min post-injection according to current clinical guidelines [20] and transferred to the test–retest images for further analysis.

### 2.6. Feature Extraction for Test–Retest Analysis

**Figure 2:**
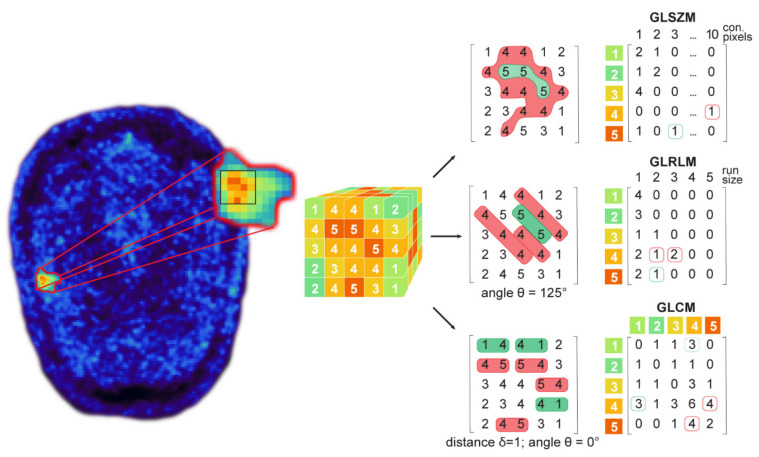
The calculation of textural features based on gray level matrices. Textural features are usually calculated on a previously segmented tumor and grouped based on the gray level matrices from which they are extracted. Features calculated from the gray level size zone matrix (GLSZM) represent connected gray level values and their associated zones or areas. The gray level run length matrix (GLRLM) represents consecutive voxels with the same intensity. The gray level co-occurrence matrix (GLCM) represents voxel pairs’ frequency of occurrences of the same intensity.

Radiomics features were calculated on the test and retest images for each VOI using the open-source package PyRadiomics (version 3.0) in Python [7]. As recommended for PET radiomics analyses, 64 bins with a fixed bin width of 0.15 were used for image discretization [5,38]. No voxel resampling was performed. All features were calculated on the original images and after applying wavelet and Laplacian of Gaussian (LoG) filter methods. In total, 1,302 radiomics features (93 original, 744 wavelet, 465 LoG features) were extracted. From the 93 original features, 18 features are first-order or intensity-based features calculated from image histograms. First-order features represent characteristics of the signal intensity values and do not provide spatial information. The remaining 75 features are texture features, or second-order features calculated from the gray level co-occurrence matrix (GLCM), gray level dependence matrix (GLDM), gray level run length matrix (GLRLM), gray level size zone matrix (GLSZM), and neighboring gray tone difference matrix (NGTDM). Texture features quantify image patterns and structures, considering spatial information (Figure 2). Shape features could not be evaluated as the shape of the VOIs was identical in the test and the retest images.

### 2.7. Statistical Analysis

FET PET feature repeatability was evaluated by the intraclass correlation coefficient (ICC) [39,40]. The ICC assesses the reliability of ratings or measurements by comparing the variability of individual ratings for the same subject to the total variation across all ratings and subjects. The ICC can range from 1 to −1, wherein 1 indicates perfect repeatability and −1 no repeatability. For the selection of the ICC type, we adhered to the guidelines provided by Koo and Li [41]. The “two-way mixed-effects model” was chosen, as the raters were not randomly selected from a wider group, and each rater individually evaluated each subject. We chose a “single measure type” because single raters’ values were used as the basis for the assessment and were not averaged. Additionally, an “absolute agreement definition” was chosen as differences in ratings for the same subject from two different raters were compared. Hence, we used the “two-way mixed-effects absolute agreement single measure” ICC, defined as,

(1) $$ICC=\frac{MS_{R}-MS_{E}}{MS_{R}+\left(k-1\right)\;MS_{E}+\;\frac{k}{n}\left(MS_{C}-MS_{E}\right)}\;,$$

where *MS_R_* represents mean square for rows, *MS_C_* mean square for columns, *MS_E_* mean square for errors, *k* number of raters/measurements, and *n* number of subjects. Features were considered repeatable if the lower and upper limits of the *ICC* 95% confidence interval were in the range of 0.91 to 1.00, not repeatable if between 0.01 and 0.90, and moderately repeatable if the upper limit was between 0.91 and 1.00 and the lower limit was in the range of 0.75 to 1.00 [3]. The ICC analysis was implemented in Python (Pingouin 0.3.6) [42].

Initially, to check the usefulness of the analysis, feature repeatability between the background VOIs and the tumor VOIs was calculated. Since the regions differ strongly in terms of tracer uptake, no reproducible features should be identified.

A hierarchical cluster analysis was performed to investigate the effect of different tumor genotypes on repeatability and assess the discriminative ability of the most repeatable features. Hierarchical clustering identifies relations in data by comparing and reordering (clustering) samples based on their similarity. The squared Euclidean distance determined the similarity between clusters and the Ward’s variance minimization algorithm was used to build individual clusters. Feature values were z-score normalized. The hierarchical cluster analysis was implemented in Python (SciPy 1.4.1) [43].

## 3. Results

Between tumor and background VOIs, no repeatable features were identified. The highest repeatability was found for the feature wavelet_HHH_firstorder_Skewness (ICC, 0.52; 95% confidence interval, 0.01–0.77).

**Figure 3:**
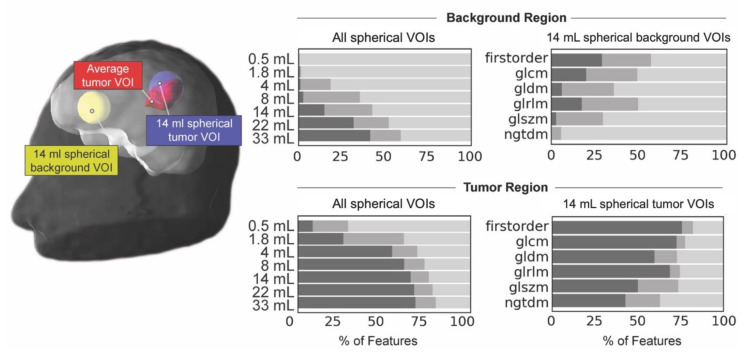
Comparison of feature repeatability for background and tumor regions. Left: Spherical tumor volumes-of-interest (VOIs) were positioned in the healthy background (yellow spherical background VOI) and the tumor volume centered on the maximum uptake (blue spherical tumor VOI). The average tumor VOI, used for most of the experiments, is presented in red. Right: Upper panels represent feature repeatability for the background region. The lower panels represent feature repeatability for the tumor region. Left panels show the percentage of feature repeatability for individual volumes. Right panels present the percentage of feature repeatability of individual feature groups for the 14 mL volume only. Features were considered repeatable if the lower and upper limits of the intraclass correlation coefficient 95% confidence interval were in the range of 0.91 to 1.00 (+, dark gray), not repeatable if between 0.01 and 0.90 (−, light gray), and moderately repeatable if the upper limit was between 0.91 and 1.00 and the lower limit was in the range of 0.75 to 1.00 (o, medium gray). glcm = gray level co-occurrence matrix; gldm = gray level dependence matrix; glrlm = gray level run length matrix; glszm = gray level size zone matrix; ngtdm = neighboring gray tone difference matrix.

In the tumor VOIs, 50% of features were repeatable, whereas only 13% in the background VOIs. In the largest spherical tumor VOI (volume, 33 mL), 67% of features were repeatable, whereas less than 26% were repeatable in tumor VOIs with a volume smaller than 4 mL (Figure 3).

To reduce the influence of tumor volume on repeatability, all following results refer to spherical tumor VOIs with a volume similar to the average tumor volume in our patient cohort (volume of spherical tumor VOI, 13.9 mL; average tumor volume, 12.5 mL). The highest fraction of repeatable features (73%) was identified for the first-order features. For textural or second-order features, the underlying gray level matrices influenced feature repeatability (GLCM, 71% repeatable features; GLRLM, 68% repeatable features; GLDM, 57% repeatable features; GLSZM, 48% repeatable features; NGTDM, 38% repeatable features).

**Figure 4:**
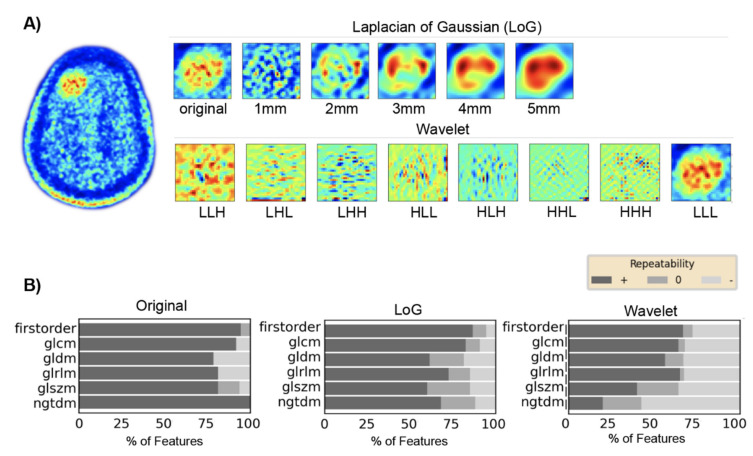
Influence of filter methods on feature repeatability. (**A**) Upper panels: Representation of tumor region as original image (left) and after Laplacian of Gaussian filtering with different sigma settings (range of sigma, 1–5 mm). Lower panels: Representation of the tumor region after wavelet filtering with individual combinations of wavelet low- (L) and high-pass (H) decompositions. (**B**) Feature repeatability in percent of individual feature groups for each image presentation. Features were considered repeatable if the lower and upper limits of the intraclass correlation coefficient 95% confidence interval were in the range of 0.91 to 1.00 (+, dark gray), not repeatable if between 0.01 and 0.90 (−, light gray), and moderately repeatable if the upper limit was between 0.91 and 1.00 and the lower limit was in the range of 0.75 to 1.00 (o, medium gray). glcm = gray level co-occurrence matrix; gldm = gray level dependence matrix; glrlm = gray level run length matrix; glszm = gray level size zone matrix; ngtdm = neighboring gray tone difference matrix.

Features calculated on unfiltered images resulted in higher repeatability (repeatable features, 87%) than on images filtered with LoG (repeatable features, 74%) or wavelet decompositions (repeatable features, 57%) (Figure 4).

**Figure 5:**
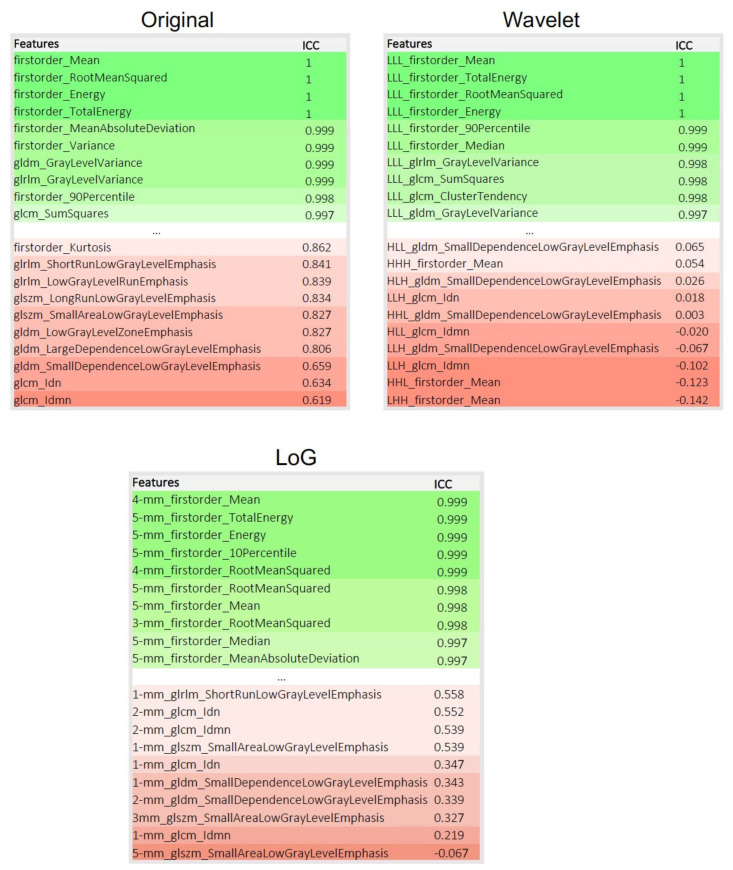
Visualization of the ten most and least repeatable FET PET radiomics features for the original (unfiltered) images, after wavelet and Laplacian of Gaussian (LoG) filtering. Feature names are shown next to the intraclass correlation coefficient (ICC) values. Calculations have been performed on the 14 mL volume spherical tumor volume-of-interest representing the average tumor volume in our study.

The ten most and least repeatable features for the unfiltered and filtered images are summarized in Figure 5.

**Figure 6:**
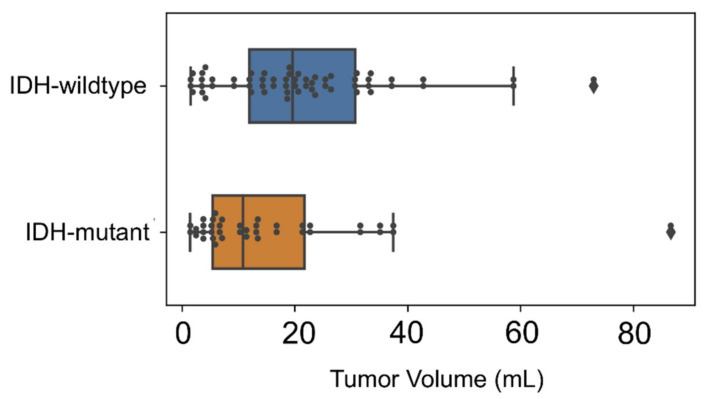
Distribution of tumor volumes in our patient cohort. Boxplots representing tumor volumes of the patient cohort. Patients with isocitrate dehydrogenase (IDH)-wildtype gliomas (blue) presented larger volumes than patients with IDH-mutant gliomas (orange).

Patients with IDH-wildtype gliomas showed higher feature repeatability compared to IDH-mutant gliomas (repeatable features, 28% vs. 16%) (Figure 6).

**Figure 7:**
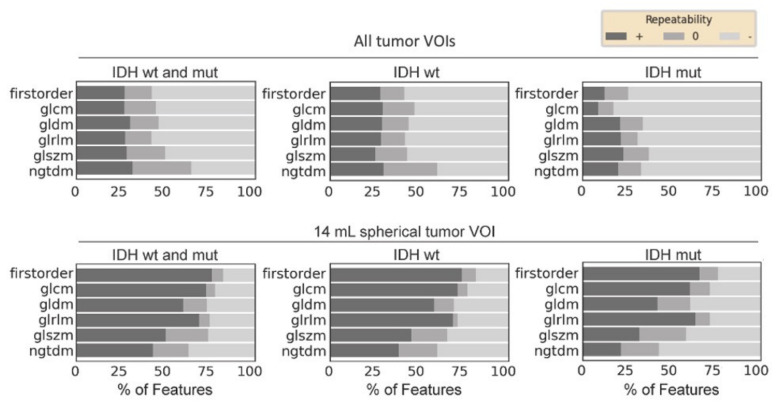
Influence of the isocitrate dehydrogenase (IDH) genotype on feature repeatability. Feature repeatability of individual feature groups in percentage shown for IDH-wildtype and IDH-mutant gliomas. Features were calculated for every sample on the complete tumor region (upper panels), or for the 14 mL spherical tumor volume-of-interest (VOI) representing the average tumor volume in our study (lower panels). Features were considered repeatable if the lower and upper limits of the intraclass correlation coefficient 95% confidence interval were in the range of 0.91 to 1.00 (+, dark gray), not repeatable if between 0.01 and 0.90 (−, light gray), and moderately repeatable if the upper limit was between 0.91 and 1.00 and the lower limit was in the range of 0.75 to 1.00 (0, medium gray). glcm = gray level co-occurrence matrix; gldm = gray level dependence matrix; glrlm: gray level run length matrix; glszm = gray level size zone matrix; IDH = isocitrate dehydrogenase; MUT = mutant; ngtdm = neighboring gray tone difference matrix; VOI = volume-of-interest, WT = wildtype.

However, this effect could be due to smaller volumes of IDH-mutant gliomas in comparison with IDH-wildtype tumors in our patient cohort (mean volume, 16.7 vs. 21.4 mL) (Figure 7).

Repetition of the analysis with a fixed, representative volume in both groups yielded comparable repeatability between IDH-wildtype and IDH-mutant gliomas (repeatable features, 63% vs. 52%) (Figure 6).

**Figure 8:**
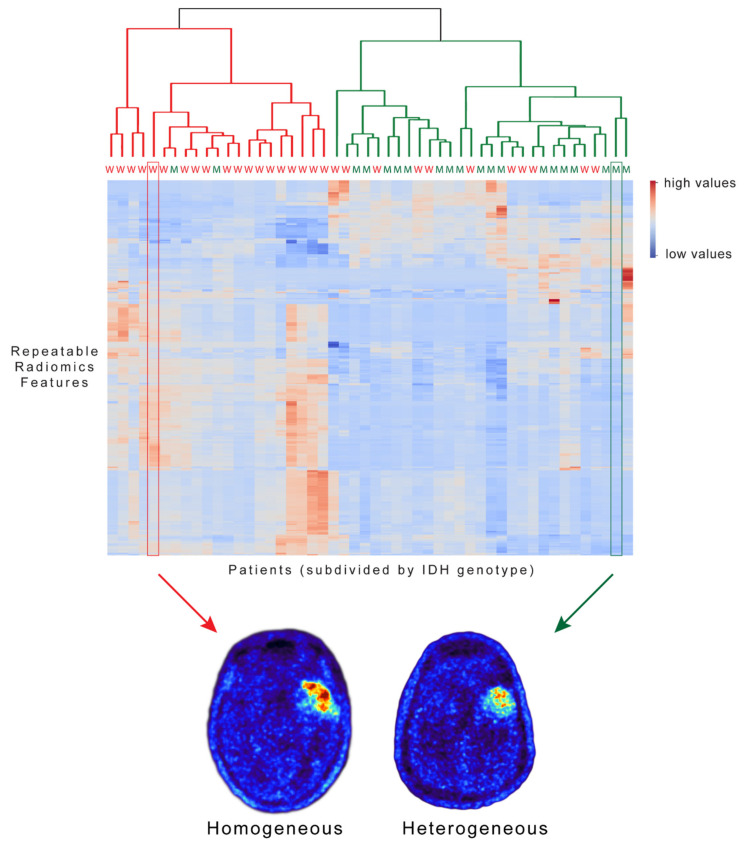
Hierarchical cluster analysis using repeatable FET PET radiomics features. Heatmap represents the absolute radiomics feature values, with higher feature values shown in red and lower values in blue. Each row represents a single feature (n = 297). Each column represents a single patient (n = 50) with a known IDH genotype (W = IDH-wildtype (red); M = IDH-mutant (green)). Features were calculated for the tumor regions. The dendrogram represents the cluster distance indicating the order in which clusters were joined.

The hierarchical cluster analysis using a subset of 297 repeatable features identified two distinct patient clusters according to the two different IDH genotypes. In cluster 1, 90% of patients had IDH-wildtype gliomas (*n* = 19/21) whereas 63% of patients in cluster 2 had IDH-mutant gliomas (19/30) (Figure 8).

## 4. Discussion

The acceptance of radiomics models and their translation into clinical routine depends on their performance in improving diagnostic accuracy, especially when conventional image evaluation leads to equivocal results. In this context, generating radiomics models and identifying reliable, repeatable, and generalizable features is essential [4].

Several studies have evaluated radiomics feature repeatability based on MRI, computed tomography (CT), and PET [44,45,46,47,48]. In this context, most studies require additional and costly patient scans prone to errors due to patient’s movements between measurements. Alternatively, various image perturbations can be used for the assessment of feature robustness [3]. Nevertheless, phantoms are often used in test–retest studies, which are not widely available, are expensive, and require special preparation. In the present study, we applied a novel dual image reconstruction method combined with a test–retest analysis that automatically enables the evaluation of feature repeatability based on routine FET PET scans with little effort. Furthermore, radiomics features with the highest repeatability can be individually determined for every study and clinical environment, which facilitates a tailored optimization of the performance and robustness of the developed radiomics models.

One main finding of the study is that robust radiomics features can be obtained from routinely acquired FET PET scans of glioma patients, which are valuable for further standardization of radiomics analyses in neurooncology. In particular, first-order features extracted from the image histogram showed the highest repeatability. This is in line with the results from an extensive systematic review on the repeatability and reproducibility of radiomics features in clinical studies [46]. Among first-order features, mean, 10th- and 90th-percentile, and energy showed the highest repeatability. Importantly, energy strongly depends on the tumor volume and might represent a difficult measure for datasets comprising strongly heterogeneous tumor volumes.

Among textural or second-order features, the parameter gray level variance (GLV), a measure of heterogeneity calculated from the GLSZM and the GLRLM, showed high repeatability (ICC 95% confidence interval, 0.91–1.00). Interestingly, also the features short zone high gray level emphasis (SZHGE) and long run high gray level emphasis (LRHGE) were found to be highly repeatable. These two parameters were also identified in a previously reported FET PET radiomics model for predicting the IDH genotype [14].

An earlier study has suggested that the frequently used textural features coarseness and contrast based on the GLCM appeared to be least stable in radiomics analyses based on [^18^F]-2-fluoro-2-deoxy-D-glucose (FDG) PET radiomics [48]. In our study, coarseness and contrast were repeatable only for FET PET tumor volumes larger than 14 mL, which could also be attributed to the use of different tracers.

Another main finding is that our results suggest a dependency of feature repeatability on tumor volume. In particular, FET PET radiomics feature repeatability increased with a higher tumor volume, which is in line with the results from a previous study using FDG PET [47]. For tumor volumes larger than 4 mL, more than 50% of features showed high repeatability. Consequently, this may be considered a threshold for reproducible results in our study and for subsequent studies. Furthermore, the lower spatial resolution in PET imaging may affect also the repeatability, especially in combination with smaller tumor VOIs.

Besides the lower spatial resolution, PET images are affected by statistical image noise caused by the random nature of radioactive decay [49]. Other sources of noise in PET include, but are not limited to, scattered and random coincidences, modes of attenuation correction, scanner electronics, and reconstruction algorithms [50]. Therefore, a reduction in statistical image noise by applying image filters may result in increased feature repeatability [47,51]. Even though it has been suggested that image filtering has only a small effect on the repeatability of FDG PET textural features [48], we observed a definite link between repeatability and image filtering. For the average tumor volume in our study, some filter settings resulted in a higher repeatability whereas other settings reduced feature repeatability compared to features calculated on unfiltered images. Thus, the influence of image filters on feature repeatability strongly depends on the type of filter and the filter kernel. Additionally, image filters bear the general risk of losing or altering the image information.

Finally, we observed in our study that the repeatability between IDH-wildtype and IDH-mutant gliomas seems to be comparable (repeatable features, 63% vs. 52%). Since radiomics features may be related to the underlying disease [2,3], feature repeatability could also be affected by different brain tumor subtypes. Here, the IDH genotype, one of the key molecular alterations in gliomas, does not influence feature repeatability in our study. Moreover, the identified robust FET PET radiomics features (*n* = 297) in our study were able to differentiate between IDH-mutant and IDH-wildtype glioma patients. This finding is in line with an earlier study demonstrating the non-invasive prediction of the IDH genotype using FET PET radiomics with high accuracy [14]. While IDH-wildtype gliomas were correctly classified with a high accuracy of 90%, the identification of IDH-mutant gliomas was inferior, with an accuracy of 63%. This could be related to the smaller tumor volumes in this group of patients compared to the IDH-wildtype gliomas.

Several limitations of the study need to be addressed. Our study investigated FET PET radiomics feature repeatability using data from a high-resolution BrainPET scanner that is not widely available. Thus, the robust features identified in our study might not necessarily be transferable to other PET scanners. However, since the developed test–retest reconstruction allows for the investigation of feature repeatability based on routine clinical scans, further studies using other PET scanners are warranted. Furthermore, the placement of large spherical VOIs onto small volumes of increased FET might affect the repeatability due to the background activity included in the analysis. Nevertheless, since the repeatability is limited in the background region, the reported repeatability may be underestimated. Importantly, although the investigated patient cohort includes a heterogenous distribution of tumor volumes, it reflects a clinically representative group of glioma patients.

## 5. Conclusions

The presented dual image reconstruction method allows the investigation of PET radiomics feature repeatability based on routinely acquired clinical PET scans. A large number of robust FET PET radiomics features could be identified, which may be useful for further standardization of radiomics analysis in neurooncology. Differences in tumor volumes affected feature repeatability, whereas the IDH genotype did not influence the results. The identified subset of robust features showed its potential for the differentiation of IDH-wildtype from IDH-mutant gliomas. Therefore, multi-center studies on FET PET radiomics feature reproducibility are warranted to improve standardization and increase the acceptance of radiomics studies.

## Figures and Tables

**Table 1:** Patients characteristics.

Patients (Total)	50
Gender	
Female	17
Male	33
Age	
Mean ± standard deviation	50 ± 15 years
Range	21–82 years
Tumor type	
Glioblastoma (IDH wt/mut)	24/2
AA III (IDH wt/mut)	4/11
A II (IDH wt/mut)	2/2
AO III	5
IDH genotype	
Wildtype	30
Mutant	20
Tumor volume	
Median	16.2 mL
Mean	12.5 mL
Range	1.4–85.2 mL

A II = WHO grade II diffuse astrocytoma; AA III = WHO grade III anaplastic astrocytoma; AO III = WHO grade III anaplastic oligodendroglioma; IDH = isocitrate dehydrogenase; mut = mutant; wt = wildtype.

## Data Availability

The data are not publicly available due to privacy and ethical restrictions.
